# Microvascular decompression in patients with hemifacial spasm

**DOI:** 10.1002/brb3.1432

**Published:** 2019-10-15

**Authors:** Xuegang Niu, Hongtao Sun, Fei Yuan, Xuyi Chen, Zhengjun Wei, Hang Wang, Jibin Ren, Jian Zhang, Weixin Li

**Affiliations:** ^1^ Institution of Neurological Trauma and Repair Characteristic Medical Center of the Chinese People's Armed Police Force Tianjin China; ^2^ Tianjin Fourth Central Hospital Tianjin China; ^3^ Department of Radiology Characteristic Medical Center of the Chinese People's Armed Police Force Tianjin China; ^4^ Logistics College of the Chinese People's Armed Police Force Tianjin China

**Keywords:** hemifacial spasm, hypertension, microvascular decompression, neurovascular compression

## Abstract

**Purpose:**

To study blood pressure alterations after microvascular decompression (MVD) surgery in patients with hemifacial spasm (HFS).

**Methods:**

A retrospective study was performed to review HFS patients who received MVD surgery between January 2014 and December 2016. Vessels that were considered to be responsible for HFS were determined by reviewing the brain magnetic resonance imaging, magnetic resonance angiography, and surgical video. Blood pressure measurements were performed 1 day before (preoperative) and 7 days after (postoperative) the MVD surgery. Pre‐ and postoperative blood pressure measurements were compared.

**Results:**

A total of 374 patients were included in the study, with 118 (31.6%) male patients, age 53.8 ± 9.9 years old, and 141 (37.7%) patients with hypertension. Systolic blood pressure had statistically significant decrease in patients with (134.5 ± 8.2–132.6 ± 9.1 mmHg, *p* = .01) or without (125.6 ± 9.1–123.8 ± 10.0 mmHg, *p* = .01) hypertension. Diastolic blood pressure only had statistically significant decrease in patients with hypertension (83.0 ± 5.8–82.0 ± 6.5 mmHg, *p* = .04). Analyses in all the study patients and in the subgroup of patients with hypertension showed that more statistically significant blood pressure reductions were observed when left‐side vessel or vertebrobasilar artery was involved.

**Conclusion:**

In patients with HFS, MVD not only decreased blood pressure in patients with hypertension but also affected blood pressure in patients without hypertension. Blood pressure reductions were more prominent when left‐side vessel or vertebrobasilar artery was involved.

## Graphical abstract

We tried to study blood pressure alterations after microvascular decompression (MVD) surgery in patients with hemifacial spasm (HFS). We found that in patients with HFS, MVD not only decreased blood pressure in patients with hypertension but also affected blood pressure in patients without hypertension. Blood pressure reductions were more prominent when left‐side vessel or vertebrobasilar artery was involved.

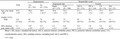

## 1 INTRODUCTION

Hemifacial spasm (HFS) is a neuromuscular disease with the clinical presentation of paroxysmal involuntary muscle contractions on one side of face (Chaudhry, Srivastava, & Joshi, 2015). The most common cause is the vascular compression of the facial nerve at its root exit zone (REZ) in the brainstem. Clinical outcomes can range from mild inconvenience to severe life disturbance due to pain and disfiguring from frequent muscle contractions (Yaltho & Jankovic, 2011). Treatments for HFS include analgesics, anticonvulsants, botulinum toxin injection to paralyze the facial muscles, and microvascular decompression (MVD) surgery to relieve the pressures on the facial nerve (Barker, Jannetta, Bissonette, Larkins, & Jho, 1996; Inoue et al., 2017; Li et al., 2016; Sindou, Leston, Decullier, & Chapuis, 2007).

Hypertension is a very common disease. A subset of patients with hypertension whose blood pressures are difficult to control despite multiple antihypertensive medication treatments are considered to have refractory hypertension. Some of these patients with refractory hypertension can have concurrent HFS (Leong, Li, Chan, & Tan, 2016). Their hypertension may be due to neurogenic causes from increased sympathetic activities from the vascular compression at the left rostral ventrolateral medulla (RVLM) and REZ of cranial nerve IX, X (Jannetta & Gendell, 1979; Naraghi et al., 1994; Sandell, Holmen, & Eide, 2013; Solar, Ceral, Zizka, & Elias, 2009). Studies have shown that MVD surgery could improve the clinical symptoms of HFS and, at the same time, could also decrease the blood pressure in patients with refractory hypertension (Barley & Ellis, 2013; Sindou, Mahmoudi, & Brinzeu, 2015). However, it is unknown whether MVD surgery only lowers the blood pressure in patients with hypertension or also alters the blood pressure in patients without hypertension. In addition, it is unknown whether patients with different responsible vessels for HFS can have different blood pressure alterations after MVD surgery.

In the current study, we report the blood pressure alterations after MVD surgery in patients with HFS. We analyzed the effects of different clinical situations, including hypertension, side of compression, and responsible vessels, on blood pressure alterations after MVD surgery.

## 2 MATERIALS AND METHOD

### 2.1 Study design and participants

The study was a retrospective study, which reviewed the patients received MVD due to HFS in our hospital during January 2014 and December 2016. The study protocol was approved by the hospital ethics committee.

Inclusion criteria were (a), diagnosis of primary HFS based on the clinical presentation and identification of the responsible vessel by brain magnetic resonance imaging (MRI), magnetic resonance angiography (MRA), or surgical operation (Rosenstengel, Matthes, Baldauf, Fleck, & Schroeder, 2012); and (b), no significant clinical improvements after conservative treatments, such as botulinum toxin injection, acupuncture, and traditional Chinese medicine. Exclusion criteria were (a), diagnosis of secondary HFS or atypical facial spasm and (b), contraindicated for MVD surgery.

### 2.2 Study protocol

Medical records were reviewed, and data, including age, gender, diagnosis of hypertension, and duration of HFS, were recorded.

The vessel that was considered responsible for HFS was determined by reviewing the brain MRI, MRA, and the surgical videos by two neurosurgeons and two radiologists. First, using the Siemens Verio 3.0 T magnetic resonance equipment, the cross‐sectional volumetric scan was performed by the 3D TOF MRA sequence, the T1_vibe_fs sequence, and the T2‐space‐iso sequence in the cerebellopontine angle region. For the three‐dimensional reconstruction of the oblique coronal plane parallel to the facial auditory nerve and the oblique sagittal plane perpendicular to the auditory nerve, the 3D TOF MRA sequence and the T1_vibe_fs sequence were used for maximum density projection to show the relationship between vessels and nerves, as well as the network of the vessels. Later on, two radiologists determined the responsible vessels based on the images of the nerve roots. Finally, two neurosurgeons fully explored the responsible vessels in the facial nerve root during the operation, and clarified the responsible vessels based on the vascular anatomy. The responsible vessels were assigned as left‐ or right‐side anterior inferior cerebellar artery (AICA), posterior inferior cerebellar artery (PICA), vertebrobasilar artery (VA), and multiple arteries (MA) including both AICA and PICA.

Microvascular decompression surgery was performed under general anesthesia with endotracheal intubation. Patients were placed in the lateral decubitus position, and the infratentorial–retrosigmoid approach was used. A 4‐centimeter skin incision was made at the affected occipital area. The scalp, muscle, and periosteum were open, and a 2 × 2 cm bone window was created by a driller and rongeur at the squamous part of occipital bone. The outer edge of the bone window was the sigmoid sinus, and the lower edge was close to the skull base. The dura mater was cut open in a "⊥" shape and suspended. Under the microscopic examination, the cerebellum was exposed and loosened in order to fully expose the facial nerve REZ area. After confirming the responsible vessel for HFS, a polyester cotton pad was used to separate the vessel and nerve at a single or multiple compression points. A biological glue was used to stick the thick vertebral artery to the petrous bone if necessary in order to ensure the complete pressure relief for the cranial nerve. The dura mater and skull were closed after complete hemostasis was achieved.

### 2.3 Outcome measurements

Blood pressure was measured 1 day before the surgery (preoperative measurement) and 7 days after the surgery (postoperative measurement). On the day of the measurement, blood pressure was checked manually three times at 6:00 a.m., 2:00 p.m., and 6:00 p.m. The mean number from these three blood pressure measurements was recorded as the blood pressure value for that day. Systolic and diastolic pressures were documented separately.

### 2.4 Statistical analysis

Numerical data were presented as mean ± *SD*, and categorical data were presented as percentages. Pre‐ and postoperative blood pressure measurements in the same patients were compared by the paired *t* test. Comparisons between different groups were performed by *t* test or chi‐square analysis when appropriate. A *p* < .05 was considered statistically significant. All the statistical analyses were performed with SPSS (version 20.0, IBM, USA).

## 3 RESULTS

### 3.1 Patient selection and baseline characteristics

Altogether, 389 patients received the MVD surgery during the study period and 374 patients were identified, with 118 (31.6%) male patients and age 53.8 ± 9.9 years old (Table 1). Patients with hypertension (141 patients, 37.7%, 58.6 ± 7.5 years old) were older than patients without hypertension (233 patients, 62.3%, 50.9 ± 10.1 years old). Numbers of patients with the right or left vessel involvement were 175 (46.8%) and 199 (53.2%), respectively. More patients were found to have AICA involvement (171, 45.7%) compared to patients with other vessel involvements (PICA, 119%, 31.8%; VA, 57%, 15.2%; and MA, 27%, 7.2%). There was no antihypertensive medication change during the pre‐ and postoperative periods.

**Table 1:** Baseline characteristics (*N* = 374)

	Total	Hypertension		Responsible vessel					
		Yes (*N* = 141)	No (*N* = 233)	Anatomical side		Vessels			
				Right (*N* = 175)	Left (*N* = 199)	AICA (*N* = 171)	PICA (*N* = 119)	VA (*N* = 57)	MA (*N* = 27)
Age, year, mean ± *SD*	53.8 ± 9.9	58.6 ± 7.5	50.9 ± 10.1*	54.4 ± 10.3	53.3 ± 9.5	51.1 ± 10.5	55.1 ± 9.1	57.2 ± 8.5	57.7 ± 8.1*
Gender, *N* (%)									
Male	118 (31.6%)	44 (37.3%)	74 (62.7%)	43 (24.6%)	75 (37.7%)	49 (28.7%)	38 (31.9%)	20 (35.1%)	11 (40.7%)
HFS, duration, year, mean ± *SD*	6.1 ± 5.3	5.87 ± 4.5	6.3 ± 5.7	6.3 ± 5.7	6.0 ± 4.8	5.8 ± 5.3	7.0 ± 5.5	5.6 ± 4.6	6.2 ± 5.4

Abbreviations: AICA, anterior inferior cerebellar artery; MA, multiple arteries, including both AICA and PICA; Mean ± *SD*, mean ± standard deviation; PICA, posterior inferior cerebellar artery; VA, vertebrobasilar artery.

* *p* < .01.

### 3.2 Alterations in blood pressure measurements before and after MVD in patients with or without hypertension

As shown in Table 2, systolic blood pressure had statistically significant decreases in patients with or without the history of hypertension, but diastolic blood pressure had statistically significant decrease only in patients with the history of hypertension.

**Table 2:** Blood pressure measurements before and after microvascular decompression in patients with or without hypertension

	Microvascular decompression			*p*
	Preoperative, mmHg, mean ± *SD*	Postoperative, mmHg, mean ± *SD*	Difference (pre – post), mmHg, mean ± *SD*	
Patients with hypertension (*N* = 140)				
Systolic pressure	134.5 ± 8.2	132.6 ± 9.1	1.8 ± 9.1	.01
Diastolic pressure	83.0 ± 5.8	82.0 ± 6.5	1.0 ± 7.0	.04
Patients without hypertension (*N* = 216)				
Systolic pressure	125.6 ± 9.1	123.8 ± 10.0	1.8 ± 9.7	.01
Diastolic pressure	78.9 ± 6.1	78.9 ± 8.1	−0.1 ± 8.1	.74

Abbreviation: Mean ± *SD*, mean ± standard deviation.

### 3.3 Alterations in blood pressure measurements before and after MVD in patients with right or left vessel involvement

Systolic blood pressure had statistically significant decrease in patients with either right vascular or left vascular involvement, with more prominent change observed in patients with left vascular involvement (Table 3). There was no statistically significant change in diastolic pressure.

**Table 3:** Blood pressure measurements before and after microvascular decompression in patients with right or left vessel involvement

	Microvascular decompression			*p*
	Preoperative, mmHg, mean ± *SD*	Postoperative, mmHg, mean ± *SD*	Difference (pre – post), mmHg, mean ± *SD*	
Right vessel involvement (*N* = 175)				
Systolic pressure	127.6 ± 10.0	126.4 ± 11.2	1.2 ± 9.0	.04
Diastolic pressure	80.4 ± 6.3	80.1 ± 8.7	0.3 ± 8.0	.29
Left vessel involvement (*N* = 199)				
Systolic pressure	130.3 ± 9.4	128.1 ± 9.9	2.3 ± 9.7	<.01
Diastolic pressure	80.6 ± 6.3	80.2 ± 6.6	0.5 ± 7.4	.24

Abbreviation: Mean ± *SD*, mean ± standard deviation.

### 3.4 Alterations in blood pressure measurements before and after MVD in patients with different responsible vessels

Statistically significant blood pressure changes were observed in patients with VA involvement (Table 4). In addition, systolic pressure also had statistically significant decrease when AICA was involved.

**Table 4:** Blood pressure measurements before and after microvascular decompression in patients with different responsible vessels

	Microvascular decompression			*p*
	Preoperative, mmHg, mean ± *SD*	Postoperative, mmHg, mean ± *SD*	Difference (pre – post), mmHg, mean ± *SD*	
Responsible vessels				
AICA (*N* = 171)				
Systolic pressure	127.2 ± 9.7	125.5 ± 10.1	1.7 ± 8.3	<.01
Diastolic pressure	79.4 ± 5.7	79.3 ± 7.6	0.1 ± 6.7	.45
PICA (*N* = 119)				
Systolic pressure	129.7 ± 8.6	129.4 ± 12.1	0.4 ± 9.8	.83
Diastolic pressure	80.6 ± 6.2	81.3 ± 8.7	−0.6 ± 8.9	.66
VA (*N* = 57)				
Systolic pressure	133.2 ± 11.5	127.1 ± 8.2	6.1 ± 10.5	<.01
Diastolic pressure	83.3 ± 7.1	79.8 ± 5.5	3.5 ± 7.3	<.01
MA (*N* = 27)				
Systolic pressure	129.1 ± 7.6	129.6 ± 9.0	−0.5 ± 10.0	.79
Diastolic pressure	80.7 ± 6.4	81.0 ± 6.0	−0.3 ± 6.6	.41

Abbreviations: AICA, anterior inferior cerebellar artery; MA, multiple arteries, including both AICA and PICA; Mean ± *SD*, mean ± standard deviation; PICA, posterior inferior cerebellar artery; VA, vertebrobasilar artery.

### 3.5 Subgroup analysis of blood pressure changes before and after MVD in patients with hypertension

In patients with hypertension, statistically significant blood pressure decrease was only observed in patients with left‐, but not right‐, side vascular involvement (Table 5). Both systolic and diastolic pressures had statistically significant decreases in patients with VA vascular involvement, and only systolic pressure was decreased in patients with AICA involvement.

**Table 5:** Blood pressure measurements before and after microvascular decompression in patients with hypertension (*N* = 141)

	Microvascular decompression			*p*
	Preoperative, mmHg, mean ± *SD*	Postoperative, mmHg, mean ± *SD*	Difference (pre – post), mmHg, mean ± *SD*	
Anatomical side				
Right (*N* = 62)				
Systolic pressure	134.7 ± 8.2	134.2 ± 10.4	0.1 ± 10.0	.98
Diastolic pressure	83.8 ± 5.3	83.6 ± 7.1	0.1 ± 7.1	.71
Left (*N* = 79)				
Systolic pressure	134.3 ± 8.3	131.1 ± 8.0	3.2 ± 8.1	<.01
Diastolic pressure	82.4 ± 6.1	80.7 ± 5.7	1.7 ± 6.8	.02
Responsible vessels				
AICA (*N* = 55)				
Systolic pressure	132.8 ± 8.1	130.8 ± 8.2	1.9 ± 7.8	.01
Diastolic pressure	81.9 ± 5.0	81.0 ± 6.4	0.9 ± 6.0	.08
PICA (*N* = 42)				
Systolic pressure	135.1 ± 6.0	136.6 ± 10.2	−2.1 ± 8.3	.10
Diastolic pressure	82.2 ± 5.1	83.5 ± 7.4	−1.7 ± 7.3	.31
VA (*N* = 30)				
Systolic pressure	139.4 ± 9.1	130.7 ± 6.9	8.7 ± 8.3	<.01
Diastolic pressure	86.6 ± 7.1	80.4 ± 5.4	6.2 ± 6.7	<.01
MA (*N* = 14)				
Systolic pressure	128.7 ± 7.2	130.6 ± 11.3	−1.9 ± 9.5	.90
Diastolic pressure	81.9 ± 4.6	83.9 ± 5.0	−2.1 ± 3.7	.06

Abbreviations: AICA, anterior inferior cerebellar artery; MA, multiple arteries, including both AICA and PICA; Mean ± *SD*, mean ± standard deviation; PICA, posterior inferior cerebellar artery; VA, vertebrobasilar artery.

## 4 DISCUSSION

Previous studies have shown that MVD surgery could not only relieve the clinical symptoms of HFS but also improve the blood pressure management in patients with hypertension (Barley & Ellis, 2013; Sindou et al., 2015). Symptomatic treatments, such as botulinum toxin injections, medicine, and acupuncture induce, did not cause significant changes in blood pressure. Our current study showed that MVD not only reduced the blood pressure in patients with hypertension, but also statistically significantly decreased the systolic blood pressure in patients without hypertension. There were more significant blood pressure reductions if the left‐side vessel or VA was involved. This was observed in all the study patients and in the subgroup of patients with hypertension.

Hemifacial spasm can cause involuntary facial muscle contractions. Hypertension is a common disease. Patients with HFS could have concurrent hypertension. Some patients with both of these disorders were considered to share the same etiology (Miller & Selman, 2012; Sindou et al., 2015). HFS was caused by the vascular compression of the facial nerve in the REZ area in the brainstem. Some patients with hypertension could have increased sympathetic tone from the vascular compression at the RVLM and cranial nerve IX, X REZ areas. Since the locations for both compressions are close together, surgical procedure to relieve the compression in the brainstem could relieve the clinical symptoms of HFS and improve the blood pressure control in hypertension. This has been shown by many previous anatomical, pathophysiological, animal, and clinical studies (Barley & Ellis, 2013; Naraghi et al., 1994; Yamamoto, Yamada, & Sato, 1991). In the current study, we demonstrated that the effect of blood pressure reduction was not only observed in patients with the history of hypertension, but also showed in patients without the history of hypertension. This might suggest that, in patients with HFS, their sympathetic tones and blood pressures are affected even if the blood pressure levels do not meet the diagnostic criteria for hypertension. MVD surgery could decrease the blood pressures in these patients without a previous diagnosis of hypertension. This has never been reported before. Clinicians should pay attention and monitor the blood pressure after the MVD surgery in patients with or without a previous diagnosis of hypertension, since both high blood pressure and low blood pressure can cause serious outcomes.

We further analyzed all the patients and the subgroup of patients with the history of hypertension. The results from these two analyses were consistent which showed that more prominent blood pressure reductions were observed in patients with left‐side vascular compression (left HFS) and in patients with VA involvement. But we have not found the other BP value alternation in the other features of HFS, as gender and the disease duration of HFS. These were found in all the study patients and in the subgroup of patients with a previous diagnosis of hypertension. The higher incidence of HFS and blood pressure alteration in patients with left‐side vascular compression might be due to the more profuse vagus nerve and C1 adrenergic nerve distribution on the left side of medulla oblongata, which could affect the afferent and efferent nerves to the left ventricles (Boogaarts et al., 2012; Granata, Ruggiero, Park, Joh, & Reis, 1985). In terms of different vessels, the diameter of VA is relatively larger and the blood flow in VA is more dynamic compared with other intracranial arteries. In addition, the VA is located in the narrow space of the posterior fossa. All of these could contribute to its compressions to the adjacent nerves and cause clinical symptoms of HFS and increase blood pressure (Dou et al., 2015; El Refaee et al., 2013). AICA originates from the basilar artery and is relatively mobile in the narrow posterior fossa, which may cause neurovascular compression and lead to HFS and hypertension (Dou et al., 2015). MVD surgery could relieve the neurovascular compression and improve the clinical symptoms of HFS and blood pressure measurements.

In addition to affecting the cardiovascular function, brainstem medulla and pons have important structures to regulate vital physiological activities, including respiration, consciousness, swallowing, bladder control, and sleep cycle. There were previous reports that MVD surgery could treat hemiplegia, type II diabetes, apnea, dysphagia, and intractable hiccups (Hoffman & Stiller, 1995; Jannetta, Fletcher, Grondziowski, Casey, & Sekula, 2010; Nakahara et al., 2014; Ren et al., 2017; Saito, Hatayama, Kon, Nakamura, & Sasaki, 2016). Our current study showed that MVD surgery could affect blood pressure in patients without the history of hypertension. The MVD surgery could also alter the functions in other physiological organ systems. The evaluation of outcomes of MVD surgery should not only limit to the clinical symptoms of HFS and the cardiovascular system, but also include other physiological systems, such as respiratory function, blood glucose control, and sleep cycles. Patients who receive MVD surgery could have alterations in these organ systems in addition to symptoms relief from HFS and blood pressure changes. Careful and frequent postoperative monitoring is required for these patients.

It was suspected that symptomatic treatment, such as botulinum toxin injections, could decrease blood pressure. As a retrospective study, we did not study botulinum toxin injection in this study. We also did not have accurate records on botulinum toxin injection on every patient. We will consider to address this question in future investigations.

Limitations in the current study included single‐center study, retrospective biases, a small number of patients in the subgroup analysis, and short follow‐up time period. In addition, various factors might influence the accuracy of blood pressure measurements during the preoperative period in this retrospective study. Future prospective studies with a large sample size and long observation time are required to confirm our findings.

## 5 CONCLUSION

In patients with HFS, MVD surgery could not only decrease the blood pressure in patients with hypertension but also affect systolic blood pressure in patients without hypertension. This blood pressure‐lowering effect was more prominent when the responsible vessel was on the left side and when VA was involved.

## CONFLICT OF INTEREST

None declared.

## AUTHOR CONTRIBUTION

Hongtao Sun designed the study, Xuegang Niu and Fei Yuan prepared the manuscript, Zhengjun Wei and Xuyi Chen collected the data, Hang Wang and Jibin Ren searched the references and made data analysis, and Jian Zhang and Weixin Li polished and revised the manuscript. All the authors approved the final version of this manuscript.

## Data Availability

The datasets supporting the conclusions of this article are included within the article.
